# Pregnancy Outcome in Relation to Treatment of Murine Typhus and Scrub Typhus Infection: A Fever Cohort and a Case Series Analysis

**DOI:** 10.1371/journal.pntd.0003327

**Published:** 2014-11-20

**Authors:** Rose McGready, John Antony Jude Prakash, Santosh Joseph Benjamin, Wanitda Watthanaworawit, Tippawan Anantatat, Ampai Tanganuchitcharnchai, Clare L. Ling, Saw Oo Tan, Elizabeth A. Ashley, Mupawjay Pimanpanarak, Stuart D. Blacksell, Nicholas P. Day, Pratap Singhasivanon, Nicholas J. White, François Nosten, Daniel H. Paris

**Affiliations:** 1 Shoklo Malaria Research Unit (SMRU), Mahidol-Oxford Tropical Medicine Research Unit, Mae Sot, Tak, Thailand; 2 Centre for Tropical Medicine, Nuffield Department of Medicine, University of Oxford, Oxford, United Kingdom; 3 Immunology Laboratories, Department of Clinical Microbiology, Christian Medical College, Vellore, Tamil Nadu, India; 4 Mahidol-Oxford Tropical Medicine Research Unit (MORU), Faculty of Tropical Medicine, Mahidol University, Bangkok, Thailand; University of California San Diego School of Medicine, United States of America

## Abstract

**Background:**

There is a paucity of published reports on pregnancy outcome following scrub and murine typhus despite these infections being leading causes of undifferentiated fever in Asia. This study aimed to relate pregnancy outcome with treatment of typhus.

**Methodology/Principal Findings:**

Data were analyzed from: i) pregnant women with a diagnosis of scrub and/or murine typhus from a fever cohort studies; ii) case series of published studies in PubMed using the search terms “scrub typhus” (ST), “murine typhus” (MT), “*Orientia tsutsugamushi*”, “*Rickettsia tsutsugamushi*”, “*Rickettsia typhi*”, “rickettsiae”, “typhus”, or “rickettsiosis”; and “pregnancy”, until February 2014 and iii) an unpublished case series. Fever clearance time (FCT) and pregnancy outcome (miscarriage and delivery) were compared to treatment. Poor neonatal outcome was a composite measure for pregnancies sustained to 28 weeks or more of gestation ending in stillbirth, preterm birth, or delivery of a growth restricted or low birth weight newborn.

**Results:**

There were 26 women in the fever cohort. MT and ST were clinically indistinguishable apart from two ST patients with eschars. FCTs (median [range] hours) were 25 [16–42] for azithromycin (n = 5), 34 [20–53] for antimalarials (n = 5) and 92 [6–260] for other antibiotics/supportive therapy (n = 16). There were 36.4% (8/22) with a poor neonatal outcome.

In 18 years, 97 pregnancies were collated, 82 with known outcomes, including two maternal deaths. Proportions of miscarriage 17.3% (14/81) and poor neonatal outcomes 41.8% (28/67) were high, increasing with longer FCTs (p = 0.050, linear trend). Use of azithromycin was not significantly associated with improved neonatal outcomes (p = 0.610)

**Conclusion:**

The published ST and MT world literature amounts to less than 100 pregnancies due to under recognition and under diagnosis. Evidence supporting the most commonly used treatment, azithromycin, is weak. Collaborative, prospective clinical trials in pregnant women are urgently required to reduce the burden of adverse maternal and newborn outcomes and to determine the safety and efficacy of antimicrobial treatment.

## Author Summary

Typhus is an under-recognised and under-studied public health problem in Asia. In rural areas of Southeast Asia murine and scrub typhus are probably the most common treatable cause of fever. The estimated number of scrub typhus cases in Southeast Asia, more than 1 million yearly, results in approximately 50–80,000 deaths per year. Treatment delays due to lack of appropriate diagnostics and lack of awareness lead to a substantial health and economic impact in the one of the world's most densely populated regions. Only 97 cases in pregnancy are available from the published world literature over the past 18 years. Only 82 of these had known outcomes, including two maternal deaths. The proportion of poor neonatal outcome including stillbirth, prematurity and low birth weight was high occurring in more than 40% of pregnancies, and higher when the fever clearance time was longer. While poor neonatal outcomes were observed with all antibiotics prescribed, azithromycin appeared to be associated with shorter fever clearance times but this was not statistically significant. Evidence to support the use of azithromycin is weak. The correct antimicrobial or combination for undifferentiated fever in pregnant women in Southeast Asia is unknown.

## Introduction

The true burden of scrub typhus (ST) and murine typhus (MT) in South-east Asia remains largely unknown as diagnostic tests are rarely used or available [1]. Still less is known about typhus in pregnancy with no (large scale) epidemiological studies addressing the subject in this group [2]. No data suggests that typhus occurs more or less commonly in pregnancy. Studies showing that the malaria vector *An. gambiae* mosquitoe was more attracted to pregnant women than non-pregnant women [3], have not been performed with the vectors of ST (mites) and MT (fleas). There is a scatter of small case series and reports on ST and MT [4]–[16] which tend to suggest serious repercussions for the mother and fetus, and congenital transmission has also been reported [10], [11]. There is a definitive lack of new studies or data in the published literature with a four year gap between the two latest publications [16], [17]. This a likely reflection of the difficulty of confirming the diagnosis and following a woman treated in out- or in-patients through to pregnancy outcome in the obstetric department.

The tetracycline-class of antibiotics, principally doxycycline, treat rickettsial diseases [18]. Congenital malformation has not been associated with doxycycline use [19], [20] but the class effect ascribed to tetracyclines including possible effects on the musculoskeletal development of the fetus [21] and staining of teeth in young children [22] precludes its use. While short courses of doxycycline are probably safe there is insufficient evidence to support their use as first line therapy [5], [23]. Macrolide antibiotics such as azithromycin, are safe in pregnancy and reported to be equally effective to doxycycline for the treatment of scrub typhus [24]. The aim of this study was to undertake a detailed analysis of ST and MT cases from a previously published fever cohort and of the published literature to provide further detail on pregnancy outcomes in relation to treatment.

## Methods

### Ethical clearance

For women participating in the fever cohort the patient information sheet and consent form were available in Karen and Burmese languages. Willing participants signed (literate) or provided a thumb-print (illiterate) on the consent form. Approval for the study was granted by the Oxford Tropical Research Ethics Committee, UK (#013-03) [16].

### SMRU fever cohort women

Details of the methods can be found in this previously published study on the epidemiology of fever in refugee and migrant pregnant women on the Thai-Myanmar border where scrub and murine typhus accounted for 12.3% (26/211) of fever diagnoses in 203 women [16]. Briefly, febrile (aural temperature >37.5°C) pregnant women following the antenatal clinics of Shoklo Malaria Research Unit (SMRU) were offered a fever screen. Relevant to this study is the diagnosis typhus and malaria. Typhus was confirmed by PCR [25]–[28] and/or *in vitro* isolation of *Rickettsia* spp. [29] and/or positive reference serology measured by four-fold rise in paired sera IFA [30], [31] to define acute infection as MT (*Rickettsia typhi*) or ST (*Orientia tsutsugamushi*). Malaria was confirmed by microscopy of thick and thin malaria smears, stained with Giemsa and examined under oil immersion. Smears were only declared negative after 200 fields were read.

None of the ST and MT results were available for the patient as these were initially stored and later analyzed off-site. Women were admitted and treatment was initiated according to the clinical examination and available laboratory test results. This implies patients may have been treated for other infections such as malaria because the result was immediately available and consistent with the clinical picture. ST or MT may not have treated as it was not suspected and the laboratory result was unavailable. Patients were monitored for fever clearance time (FCT) by 6-hourly aural (Genius) temperature measurement. Women followed regular antenatal care after discharge and were encouraged to deliver with skilled birth attendants where they are weighed directly after birth. Infants born at home and not weighed on the day of birth had their weight adjusted as described for this population [32]. Poor neonatal outcome was a composite measure for women whose pregnancy was sustained to 28 weeks or more of gestation and included stillbirth, prematurity and fetal growth restriction defined as a birth weight for gestational age of <10^th^ centile for the population [33].

### Additional unpublished cases at SMRU

Since Dec 2012 pregnant women at SMRU with fever have routinely been tested with the SD Bioline Tsutsugamushi from Standard Diagnostics, Korea, a rapid test, in addition to other standard fever screening tests. This rapid diagnostic test is non-specific testing IgG, IgM and IgA *Orientia tsutsugamushi* antibodies. Acute and convalescent serum was collected. Remaining serum was stored at −20°C. These samples were collected over a one year period and processed in the same laboratory as per the aforementioned fever cohort women. ST was confirmed by PCR [25]–[28] and/or positive reference serology measured by four-fold rise in paired sera IFA [30], [31]. Women were followed up in the routine antenatal service and encouraged to birth with skilled birth attendants at Shoklo Malaria Research Unit. Of 25 women with a positive rapid test there were three with a confirmed ST diagnosis and a known pregnancy outcome included for analysis.

### Pooled analysis of all previously published data

The pooling of data into a single data set includes the aforementioned fever cohort women, additional unpublished cases, and data reported from case reports or case series in 16 different publications [5]–[7], [9], [10], [12], [15]–[17], [34] (Supporting Information S1). As the population birth weight percentiles were unknown for these publications, low birth weight (<2500 g) replaced fetal growth restriction in the definition of poor neonatal outcome. Individual patient data including age, gestation at infection, days of fever on admission, FCT, treatment, maternal death, birth outcome (including miscarriage (delivery before viability set at <28 weeks for resource limited settings), delivery and unknown), birth weight and gestational age at delivery, live birth or stillbirth, birth weight and congenital normality were extracted from published studies into a data spreadsheet (Supporting Information S2).

### Analysis

Data were described using the statistical program SPSS Statistics version 20.0 for Windows (SPSS Benelux inc., Gorinchem, Netherlands). Continuous normally distributed data were described by the mean (standard deviation, range) and non-normally distributed data by the median [min-max] and comparisons used the Student's t-test or Mann-Whitney U test, respectively. The number and percentage were given for categorical data and compared using the X^2^-test with Yates' correction, or the Fisher's exact test when applicable.

Poor neonatal outcome was compared by univariate analysis to the treatment group. Given the small sample size and to avoid erroneous conclusions no modeling of poor neonatal outcome was attempted.

## Results

### SMRU fever cohort

From 2004 to 2006, 26 febrile pregnant women were identified as having evidence of rickettsial infection with confirmation by serology in 9 women, serology and PCR in 17 women (MT n = 7, ST n = 9, and both n = 1). In 4 of these cases *O. tsutsugamushi* was isolated from blood culture and all of these women were PCR and serology positive. Culture positive cases included the single patient with mixed ST and MT infection.

Note that in 5 cases there was concomitant infection with malaria. Malaria cases were all microscopic: three MT (with two concomitant *P. falciparum* and one *P. vivax* infections) and two ST with concomitant *P. falciparum*. These five patients were grouped respectively into MT and ST [16]. The patient who had dual infection with ST and MT (dynamic serology for MT, and PCR positivity for both) documented at 37^+2^ weeks^+days^ gestation could not be placed into either group (ST or MT) for analysis. Briefly she was a 33 year old with 4 previous births, a FCT of 119 hours with IV ceftriaxone and a normal birth outcome (singleton, estimated gestation 41^+3^ weeks^+days^ and birth weight of 3600 g).

Overall there were 13 women with ST, 12 with MT and one with ST and MT. The baseline demographic and obstetric characteristics have been detailed for MT and ST diagnostic groups (Table 1). There were no significant differences observed in baseline characteristics. Of note is the higher proportion (non-significant): of flea-borne MT was higher in the refugee camp than in migrants; and of smokers in the ST group.

**Table 1:** SMRU fever cohort women baseline demographic and obstetric characteristics according to the diagnostic group.

Characteristic	Murine Typhus n = 14	Scrub Typhus n = 11	P value
Refugee women, n (%)	11 (78.6)	6 (54.5)	0.389
Age, years, mean±SD [min-max]	27±6 [16–36]	27±7 [17–38]	0.813
Gravidity, median [min-max]	2 [1–10]	3 [1–12]	0.541
Parity, median [min-max]	1 [0–5]	2 [0–9]	0.416
Primigravida, n (%)	3 (21.4)	2 (18.2)	1.000
Smokers, n (%)	5 (35.7)	6 (54.5)	0.435
Gestational age, weeks^+days^, median [min-max]	21^+5^ [7^+0^–39^+5^]	24^+4^ [6^+1^–38^+1^]	0.869
First Trimester, n (%)	4 (28.6)	3 (27.3)	1.000

#### Clinical features of SMRU fever cohort

Clinical features apart from eschar and rash (non-significant) did not distinguish MT and ST (Table 2). Women in the ST group had a longer history of fever before presentation but this was not significant (Table 2). There were no significant differences observed for hematocrit or complete blood count that might help distinguish MT from ST. Although platelet count was significantly lower in the scrub typhus group this included two women with *P. falciparum* which is known to depress the platelet count more than in *P. vivax* [35] in this population (Table 2).

**Table 2:** SMRU fever cohort women clinical features and complete blood count on admission.

	Murine Typhus n = 14	Scrub Typhus n = 11	P value
Days fever, median	3 [1–7]	5 [1–10]	0.113
>3 days fever, n (%)	4 (28.6)	9 (63.6)	0.116
Temperature °C, median	38.5 [38.0–39.9]	38.3 [37.6–39.5]	0.476
BP systolic, mmHg	100 [90–120]	100 [80–110]	0.501
BP diastolic, mmHg	63 [60–80]	60 [60–80]	0.561
Headache, n (%)	13 (92.3)	11 (100.0)	1.000
Muscle pain, n (%)	9 (64.3)	8 (72.7)	1.000
Joint pain, n (%)	7 (50.0)	9 (81.9)	0.208
Anorexia, n (%)	12 (85.7)	9 (81.8)	1.000
Nausea/vomiting, n (%)	5 (35.7)	5 (45.5)	0.697
Dizziness, n (%)	5 (50.0)	8 (72.)	0.414
Eschar, n (%)	0	2 (18.2)	0.183
Rash, n (%)	0	2 (18.2)	0.183
Splenomegaly, n (%)	2 (14.3)	2 (18.2)	1.000
Hepatomegaly, n (%)	4 (28.6)	3 (27.3)	1.000
**Complete blood count**			
Haematocrit %∧	31.2±5.2 [21.7–38.5]	30.4±4.8 [24.2–37.9]	0.679
WBC count ×10^9^/L∧	7.7±4.5 [3.5–18.9]	8.0±3.1 [3.8–13.3]	0.852
Neutrophil %∧	79.7±7.0 [69.9–91.1]	74.7±9.5 [55.1–89.2]	0.217
Lymphocyte/monocyte %∧	16.1±5.8 [7.5–26.3]	19.7±8.0 [8.4–32.5]	0.280
Mixed %∧	4.2±2.4 [1.4–8.3]	5.6±3.5 [2.4–12.4]	0.340
Platelet count ×10^9^/L ∧	169±79 [58–280]	108±50 [44–206]	**0.047**

∧ mean±standard deviation [min-max].

WBC (and differential) and platelet count available for 10 and 11 women only.

#### Fever clearance times [FCTs] and treatment of SMRU fever cohort

Even in this single-center study there was a wide range of treatments prescribed to these cases and the wide variation in FCT. The overall median [range] FCT for MT and ST was 40 [6–260] and 42 [16–159] hours with no statistically significant difference between the groups, p = 0.443. There were three women in the MT group who received supportive therapy alone and who all had spontaneous resolution of fever at 69 [7–80] hours. Women with concomitant malaria did not receive antibiotics as they were all treated with antimalarials and their fevers cleared. Ceftriaxone treated patients had longer fever clearance times but ceftriaxone has preferentially been used for pregnant women who appear more clinically unwell but without apparent gynecological tract infection in this setting.

The FCTs were: 25 [16–42] hours with azithromycin monotherapy (n = 5); 34 [20–53] hours with antimalarials (n = 5); and 92 [6–260] hours with other (or no) antibiotics (n = 16). FCT was significantly lower for azithromycin monotherapy versus other (or no) antibiotics, p = 0.029; but not significantly different for azithromycin monotherapy versus antimalarials p = 0.347 nor for antimalarials versus other (or no) antibiotics p = 0.069. FCT (hours) was weakly correlated to days of fever at presentation (MT and ST pooled): R^2^ = 0.161, p = 0.066.

#### Pregnancy outcomes of SMRU fever cohort

One woman with MT was lost to follow-up before the outcome of pregnancy was known and amongst those with typhus and a known outcome 12% (3/25) miscarried (Table 3); two of whom were malaria coinfected. Of the delivered infants 14.3% (3/21) were stillborn: one ST case (with an eschar) treated with azithromycin; and one ST, and one MT, both treated with ceftriaxone. All three stillborn infants had signs of growth restriction before the infection; and while syphilis and maternal hypertension were ruled out, two women smoked and one stillbirth resulted from placental abruption. The proportion of live born infants that were small for gestational age, 22.2% (4/18), was high and not significantly different between the groups (Table 3). The proportion of poor neonatal outcome was 33.3% (4/12) for MT and 44.4% (4/9) for ST, p = 0.673. For all women with rickettsial infections, including the MT and ST case, the proportion of poor neonatal outcome was 36.4% (8/22). Amongst the three malaria coinfected cases none had a poor neonatal outcome.

**Table 3:** SMRU fever cohort women pregnancy outcome by diagnostic group.

	Murine Typhus n = 14	Scrub Typhus n = 11	p
Outcome unknown, n (%)	1 (7.1)	0	1.000
**Known outcome**	**n = 13**	**n = 10**	
Abortion, n (%)	1 (7.7)	2 (18.2)	0.576
**Delivered infants**	**n = 12**	**n = 9**	
Stillbirths, n (%)	1 (8.3)	2 (22.2)	0.553
Congenital abnormalities	0	0	
Mean Gestational age, weeks^+days^	38^+5^±2^+1^ [34^+2^–41^+5^]	37^+6^±1^+3^ [36^+0^–40^+3^]	0.312
Proportion premature, n (%)	1 (16.7)	2 (22.2)	1.000
**Live born, weighed**	**n = 11**	**n = 7**	
Birth weight g, mean±SD [min-max]	2722±484 [1770–3260]	2949±651 [2200–2949]	0.446
SGA (<10^th^ Centile), n (%)	2 (18.2)	2 (28.6)	1.000
Poor neonatal outcome^a^	4/12 (33.3)	4/9 (44.4)	0.673
**Attended 1 month visit**	**n = 9**	**n = 6**	
Infants, alive at 1 month, n (%)	9 (100.0)	6 (100.0)	1.000

SD = standard deviation; SGA = small for gestational age.

a Poor neonatal outcomes includes stillborn, premature and/or SGA.

The days of fever reported at presentation and FCT for all women with typhus, including the case of MT+ST, were not significantly different for poor (n = 8) and normal (n = 15) pregnancy outcome: days of fever were 4 [1–7] and 3 [1–10] days, p = 0.557, and FCT were 86 [25–260] and 42 [6–144], p = 0.183, respectively.

### Additional unpublished cases SMRU

From Dec 2012 until Feb 2014 there were 22 febrile pregnant women with a positive SD Bioline Tsutsugamushi RDT and serum samples available for further diagnostic testing. Of these women only three had a 4-fold rising ST IgM titre (two 1∶400 to 1∶1600 and one 1∶1600 to 1∶6400) and none had a positive ST PCR result. All 3 women delivered term, live born, normal infants one of which was of low birth weight (treated with paracetamol and fever had cleared by the time the lab result was available). Treatment in the other two women was azithromycin in one case based on protocol, and multiple antibiotics for the other case: initially ciprofloxacin for suspected pyelonephritis, two days later erythromycin for suspected exacerbation of asthma and one day later azithromycin based on the positive RDT result with rapid fever clearance, <24 hours, once this drug was commenced.

### All published data

Over an 18 year period, from 1992 to 2014 there were 97 women with a diagnosis of MT or ST (or both) in pregnancy including the 26 reported from the SMRU fever cohort [16] described above, the 3 unpublished cases, and 85 in peer reviewed publications [5]–[7], [9], [10], [12], [15]–[17], [34] (Supporting Information S1). The number of cases (per publication) included 42 (n = 1), 26 (n = 1), 9 (n = 1), 5 (n = 2), 2 (n = 2) and 1 (n = 3), from five different countries including 52 from India, 30 from the Thai-Myanmar border, 11 from Korea, and 1 from Taiwan. The three unpublished cases were also from the Thai-Myanmar border. Reported diagnostic methods were predominantly serology-based, which included Weil-Felix and IFA testing, and few were paired titrations and/or PCR (Supporting Information S1).

#### Clinical features all published data

The women had a median [range] age of 25 [16–38] years (n = 97), gestation at presentation of 25^+4^ [6^+0^–39^+5^] weeks^(+days)^ (n = 96) and 6.5 [1–25] (n = 88) days of fever.

#### Fever clearance times [FCTs] and treatment of all published data

There were more than 20 different antibiotic regimens prescribed to these women, with most women receiving azithromycin alone 66.3% (61/92), or in combination with other antibiotics, including ciprofloxacin, ceftriaxone, ertapenem, and piperacillin plus tazobactam, presumably in patients with more severe illness.

While the highest proportion of women with short fever clearance time was observed in the azithromycin group there is no control of this data for disease severity, treatment delay, stage of the pregnancy, or intercurrent illness (Table 4).

**Table 4:** Fever clearance times (hours) from all published data according to the treatment group.

Fever clearance time hours	No azithromycin n = 30	Any azithromycin^a^ n = 52	Azithromycin combination^b^ n = 9^c^	Total n = 91
<24	5 (29.4)	12 (70.6)	0	17
24–72	13 (22.8)	37 (64.9)	7 (12.3)	57
≥72	12 (70.6)	3 (17.6)	2 (11.8)	17

a azithromycin (single dose/longer course).

b combinations included piperacillin plus tazobactam n = 4; ceftriaxone n = 3; ertapenem n = 1; ciprofloxacin and erythromycin n = 1.

c not available for one women who died before fever clearance.

#### Pregnancy outcomes of all published data

Of the 97 pregnancies there were 15.5% (15/97) where the outcome of pregnancy was unknown. Of the 82 remaining women there were 2 (2.4%) maternal deaths, both in India and with a long history of fever (8 and 9 days). One woman died within 21 hours of admission and the other after 3 days (her preterm neonate also died). There were 17.3% (14/81) of pregnancies ending in miscarriage before 28 weeks mostly from first trimester infections 71.4% (10/14). Of the 67 women with deliveries 15.2% (10/66) had stillbirths, 32.8% (20/61) preterm births, 33.3% (16/48) low birth weight and at least one of these events i.e. a poor neonatal outcome occurred in 41.8% (28/67). The combination of poor neonatal outcome and miscarriage i.e. poor pregnancy outcome, for infection in trimester 1, 2 and 3 was 62.5% (10/16), 42.9% (12/28) and 54.1% (20/37), p = 0.798, demonstrates no linear trend.

There was no significant difference in poor neonatal outcome according to the source of data collection, or with MT and ST groups (Table 5). The proportion of poor neonatal outcome increased with longer FCTs but this was not significant. Importantly, azithromycin, the current treatment of choice in pregnancy, was not significantly associated with better neonatal outcomes (Table 5).

**Table 5:** Delivered infants from combined case series according to neonatal outcome.

Variable		Neonatal outcome		
		Normal n = 39	Poor n = 28	p value
Data Source	Fever cohort	14 (63.6)^b^	8 (36.4)	
	Case series	22 (55.0)	18 (45.0)	
	Case report	3 (60.0)	2 (40.0)	0.641
Rickettsial group^a^	MT	8/12 (66.7)	4/12 (33.3)	
	ST	31/55 (56.4)	24/55 (43.6)	0.748
Fever clearance time	<24	13 (81.2)	3 (18.8)	
hours	24 to <72	19 (55.9)	15 (44.1)	
	> = 72	6 (46.2)	7 (53.8)	0.050^c^
Azithromycin	No	14 (53.8)	12 (46.2)	
	Yes	25 (62.5)	4 (37.5)	0.610

Data are n (%).

a Except the case with ST and MT n = 38.

b Includes the 3 cases of ST and malaria.

c Chi-squared for trend.

## Discussion

Definitive conclusions about the effects of rickettsiosis in pregnancy are not possible given the dearth of available evidence in the global literature. The low number of published cases from the past 18 years, amounting to 87 with a known pregnancy outcome and only 67 with a gestation of at least 28 weeks gestation, strongly suggests that scrub typhus and murine typhus is severely under recognized. In view of the 2.4% maternal mortality and the poor pregnancy outcome in survivors reported here, there is an urgent need to improve access to diagnosis and treatment. The performance of the currently available tests is not satisfactory and represents a major barrier. The one year field experience with scrub typhus RDT in pregnancy at SMRU has led to the abandonment of the test and also highlights the risk of over diagnosis of scrub typhus in endemic settings.

If rickettsial infection does not result in miscarriage the outcomes for the neonate are not encouraging. In the SMRU fever cohort where population data collected during the same period is available, typhus appeared to result in a higher proportion of preterm birth (14.3%) and birth weight below the 10^th^ centile (22.2%) than reported for malaria during pregnancy (7.3% (58/794) and 17.0% (88/519) respectively) [33].

This was also highlighted by the south India case series of fever before delivery where pregnancy loss with scrub typhus was significantly higher than observed from routine obstetric data at the same hospital: 33% vs 2.8%; P<0.001 [17].

The pathogenic mechanisms associated with adverse pregnancy outcomes in rickettsial infection are unknown. In malaria the predominant pathology is monocyte infiltration in the placental intervillous space, but whether this is similar in tyhus is unknown. The rickettsia and *orientia*-associated vasculopathies result from either direct endothelial infection and/or an inflammatory monocyte-dominant perivascular infiltration [36]–[38]. The consequences of endothelial damage in murine typhus and an inflammation-associated pro-coagulant state in scrub typhus are suggestive of a predominantly circulatory impediment, possible due to thrombotic occlusion and or coagulopathy [39]. This contrasts with the disease observed in the SMRU fever cohort where mild, self-limiting, flu-like illness, without signs of organ dysfunction such as jaundice and renal insufficiency, was observed [40]. Indeed three women all with MT made a spontaneous recovery without antibiotics. There were no convincing clinical or complete blood count features that helped differentiate ST from MT [41], [42] or suggested that MT was a milder illness than ST in pregnancy as described previously in children (Table 2) [42]. Eschar was barely mentioned in any of the reports although it can be a valuable diagnostic clue for ST, if recognized by clinicians [1].

The range of treatments given to pregnant women was wide and consistent with the difficulty of making a clinical diagnosis of ST and MT with a non-specific early phase of the disease [43]. It reflects the confusion that undifferentiated illness can cause in remote and rural areas where access to fever diagnostics is very limited. In response to this problem the guidelines at SMRU now include azithromycin as an empirical first line therapy for pregnant women in undifferentiated fever cases but since azithromycin was not associated with improved neonatal outcome this might not be the right choice? FCT has classically been used as one of the markers of an efficacious treatment of ST and MT [44] and has served to advocate for azithromycin as treatment in pregnant women with scrub typhus in Thailand [8] and Korea [5], as well as its safety profile in first trimester [8]. The antimicrobial armamentarium is limited for this disease in pregnancy. Doxycycline is inexpensive and the drug of choice in non-pregnant patients for rickettsial illness and the shortest described treatment course is at least 3 days as well as having short fever clearance times [45]. Doxycycline can be used in pregnancy, if no alternative is available and there is no other contraindication [46], which is a real case scenario in much of the rural tropics, in addition to it being a cheaper alternative than azithromycin. Most treatment efficacy studies of typhus have been limited to a short follow-up of 28 days but like malaria [47] a longer duration of follow-up is likely to be required to monitor for relapse in pregnant women and further pharmacokinetic studies are required [48]. While rifampicin monotherapy with doses of 900 mg and 600 mg (mean fever clearance times 22.5 and 27.5 hours, respectively) led to shorter fever clearance times than doxycycline monotherapy (mean fever clearance time 52 hours) in northern Thailand [49] prolonged fever clearance times were observed with the combination of rifampicin and doxycycline. A study on rifampicin for brucellosis which co-administered doxycycline observed reduced drug concentrations of doxycycline and was associated with treatment failure or relapse [50]. Rifampicin and azithromycin can decrease the level or effect of azithromycin probably by an interaction with P-glycoprotein [MDR1] transported which effects how azithromycin is eliminated from the body [45].

The usefulness of antimalarials to treat obligate intracellular bacteria is biologically plausible and appreciated only because the SMRU fever cohort explicitly aimed to investigate comprehensively the cause of fever by offering a battery of tests, identifying truly coinfected women [16]. If antimalarials are effective in the treatment of rickettsia, it may in part explain why there remains a paucity of diagnosis of rickettsia in areas with malaria. Antimalarials should be tested *in-vitro* for their effectiveness against *Orientia* and *Rickettsia* species. Thailand has been highly geared to early diagnosis and treatment of malaria over the past 4 decades and the problem of rickettsial illness may become more overt as malaria continues to decrease [51]. It needs to be noted that even if scrub typhus was diagnosed and treated as effectively as malaria, the incidence would likely not be affected since humans are dead-end hosts and immunity is short lived [2].

Many infections that predominate in low-income countries such as malaria, tuberculosis, hepatitis and rickettsioses, fail to be included as causes of maternal and neonatal mortality in part due to the difficulty of making the diagnosis and weakness in data collecting systems [52]. Indeed one of the maternal deaths in the series presented here, without PCR diagnostics, would have been classified as an indirect maternal death from sepsis as she died from multi-organ failure [53]. While maternal mortality from sepsis is reported to be on the decrease with recommendations for improved sanitation, death from ‘sepsis’ in tropical countries may be more complex than it first appears due to undiagnosed tropical neglected diseases [52]. Likewise neonatal death from preterm birth and stillbirth requires a significant investment to elucidate the true cause in tropical countries because a first trimester infection may impact many months down the line.

### Limitations

The main limitations of this study are the use of retrospective data subject to reporting bias and the weak serology-based rickettsial diagnostics of the case series [54]. Despite this the proportion of poor neonatal outcome was similar in the fever cohorts with more reliable confirmation including PCR (Table 5). Only real-time diagnostic capacity, classification of disease severity and standardized approaches to treatment in prospectively followed pregnancies will allow clarification of the findings [55]. Given that typhus represents the leading cause of treatable undifferentiated fever in Southeast Asia [56], [57], and that its regions of high endemicity are among the most populous areas in the world, the need for prospective fever-in-pregnancy studies to estimate the burden and best treatment of disease in this group is obvious. Asymptomatic infection is also a high possibility and the implications for pregnancy unknown. Theoretically azithromycin is the drug of choice in pregnancy but the worldwide data collated here only provides support for level-4 evidence for the use of this drug and this must improve.

### Conclusion

The data presented here highlights the potential implications and severity of this easily treatable infection and demonstrates the inexorably slow pace of improvement in our understanding of rickettsial illness in pregnancy: less than 100 women with a known pregnancy outcome in 18 years. Progress towards the 2015 countdown to Millenium Development Goals to improve maternal, newborn and child survival will not be met in relation to scrub and murine typhus. Evidence to support the use of azithromycin is weak. The correct antimicrobial or combination for undifferentiated fever in pregnant women in South-east Asia is unknown.

## Supporting Information

Supporting Information S1 Summary of published data on murine typhus and scrub typhus, treatment and pregnancy outcome.
(DOC)

Supporting Information S2 Excel spreadsheet of all extracted variables from published trials.
(XLS)
