# Supplementary material for: Pregnancy Outcome in Relation to Treatment of Murine Typhus and Scrub Typhus Infection: A Fever Cohort and a Case Series Analysis
Source: PLoS Negl Trop Dis. 2014 Nov 20;8(11):e3327. doi: 10.1371/journal.pntd.0003327 (PMC4238995; doi:10.1371/journal.pntd.0003327)
Supplement: Supporting Information S1 — Summary of published data on murine typhus and scrub typhus, treatment and pregnancy outcome. (DOC) [file pntd.0003327.s001.doc]

**Table 1. Published data on murine typhus, treatment and pregnancy outcome**

[search terms “*Murine typhus*”, “*Rickettsia typhi*” and “*pregnancy*”]

| **Year**  **First Author** | **Publication type (n)** | **Location** | **Age (years)** | **EGA**  **(weeks)** | **Clinical course** | **Diagnostics** | **Treatment** | **Pregnancy outcome** |
| --- | --- | --- | --- | --- | --- | --- | --- | --- |
| 1992  Graves et al | Case report  n=1 | QLD, Australia | 17 y.o. | Not avail. | viral-like illness, spotted rash, fever and headache | Serology: Proteus OX19 (Weil-Felix); IFA *Rickettsia typhi* | Erythro | Normal term infant |
| 2007  Koliou et al | Case report  n=1 | Larnaca, Cyprus | 30 y.o | 30 | Spotted rash, fever and headache, chills | Serology: IFA *Rickettsia typhi* | Erythro | Normal term infant |
| 2010  Gutierrez et al | Case report  n=1 | Texas, USA | 26 y.o | 26 | Fever and headache, chills, myalgia, RUQ pain | Serology: Proteus OX19 (Weil-Felix), IFA *Rickettsia typhi* | Ampi | Normal term infant |
| 2010  Jolley et al | Case report  n=1 | California, USA | 33 y.o | 32^+5^ | Fever and headache, chills, myalgia, | Serology: IFA *Rickettsia typhi* | Azithro | Normal term infant |
| 2010  McGready et al | Cohort  n=11 | Tak Province, Thai-Myanmar border | Mean 26yrs  [16-36] | 23+2  [10^+4^ - 39^+5^] | Fever and headache, myalgia, anorexia | Serology: IFA *Rickettsia typhi* and/or PCR | Briefly in , detailed in current manuscript | Briefly in , detailed in current manuscript |
| 1997  Anstey et al | Serological cross-sectional survey n=150 | Dar es Salaam, Tanzania. | Mean 23.3 y.o. [16-40] | Not avail. | Not unwell  (Seroprevalence 28%) | IFA *Rickettsia typhi* | Not applicable | Not reported |

*Abbreviations: EGA estimated gestational age in weeks; Ampi ampicillin; Azithro azithromycin; Erythro erythromycin*

**Table 2. Published data on scrub typhus, treatment and pregnancy outcome**

[search terms “scrub *typhus*”, “*Orienta tsutsugamushi*” and “*pregnancy*”]

| **Year**  **First Author** | **Publication type (n)** | **Location** | **Age**  **(years)** | **EGA**  **(weeks)** | **Clinical course** | **Diagnostics** | **Treatment** | **Pregnancy outcome (other)** |
| --- | --- | --- | --- | --- | --- | --- | --- | --- |
| 1981  Shirai et al | Sero-epidemiological  n=111 | Kuala Lumpur, Malaysia | Not stated | delivery | Not unwell | Serology: Indirect IFA *R. tsutsugamushi* | Not applicable | Not available  29% mothers IgG+ve  No IgM+ve in cord blood ie. No evidence fetal infection) |
| 1997  Suntharasaj et al | Case report  n=1 | Songkhla,  Thailand | 31 | 34 | Fever and headache, chills, dry cough | Serology: Proteus OX19 (Weil-Felix) and to *Rickettsia typhi* by IFA | Erythro | Suspected transplacental infection; Preterm 34wks; C.section, microcephaly, encephalomalacia |
| 1992  Tsui et al | Case report  n=1 | Yi-Lan County, Taiwan | Not avail. | Not avail. | fever and headache | Not available | Minocycline | Normal term infant |
| 1998  Choi et al | Case report  n=2 | Chung-nam, Korea | 27 | 19 | fever and headache, eschar | Serology: *O. tsutsugamushi*  (passive hemagglutination test) | Azithro | Healthy |
|  |  |  | 37 | 24 | Fever, macular rash, eschar |  | Azithro | Healthy |
| 1999 Watt et al | Case report  n=2 | Chiang-Rai Thailand | 26 | Early 1st trimester | Fever, cough, hearing loss, lymphadenopathy | Positive Immunoblot dipstick test (Dip-s-Ticks; Integrated Diagnostics, Baltimore, Md.) or IgM | Azithro | Complete abortion |
|  |  |  | 30 | 26 | Fever, cough, hearing loss, conjunctival suffusion | ≥1:400 and/or IgG ≥1:1,600 indirect immunoperoxidase assay | Azithro | Unknown |
|  |  |  |  |  |  | *Table 2 continues next page….* | | |
|  |  |  |  |  |  |  |  | Table 2 continued… |
| **Year**  **First Author** | **Publication type (n)** | **Location** | **Age**  **(years)** | **EGA**  **(weeks)** | **Clinical course** | **Diagnostics** | **Treatment** | **Pregnancy outcome (other)** |
| 2003  Mathai et al | Case report | Vellore, India | 17 | 28 | Fever, cough, chills, breathless | Weil Felix or Indirect IFA | Cipro | Stillbirth |
|  | n=5 |  | 20 | Not avail. | Fever |  | Cipro | Stillbirth |
|  |  |  | 24 | 30 | Fever and headache, chills, vomiting, dysuria, expectorant cough |  | Penicillin,  Genta & CQ prior admit then Cipro & Ampi then Chloram | Low birth weight |
|  |  |  | 32 | 16 | Fever, eschar, lymphadenopathy |  | Cefurox | 470 g Mid-trimester abortion (Reported as Stillbirth) |
|  |  |  | 21 | 11 | Fever, chills, dry cough, vomiting, myalgia |  | Cipro 1D, amikacin 2D, ceftx 5D prior admit | Abortion |
| 2004 PhuPong et al | Case Report  n=1 | Chiang-Rai Thailand | 33 | 29 | Fever and headache, chills | Scrub typhus IFA IgG > 1:6400; IgM < 1:400. | Chloram | Preterm labour 29 wks neonatal death from RDS |
| 2006  Kim et al | Case Series  n=9 | Daejeon, Republic of Korea | 30  [22-34] | 21  [10-30] | Fever and headache (n=9), myalgia (n=7) | Paired Indirect IFA IFA *R. tsutsugamushi* | Azithro  (1 case + ceftx) | 8 reported births, term healthy mean bw 2.999 [1.8-3.7]kg |
| 2009  Maharjan et al | Case series  n=5  2003-2006 | Shimla, Himachal Pradesh, India | 22  [18-26] | 23 [16^+2^- to 31^+5^] | Fever (n=5) and headache (n=4), vomiting (n=4) | Single Weil Felix and PCR (n=5 positive) | Azithro & Ceftx (died) Azithro (n= 3) Doxy& ceftx | 2 Unavailable; 1 fetus *in-utero* when mother died; 1 PTB, LBW neonatal death; 1 NVD, healthy |
| 2010  McGready et al | Cohort  n=9 | Tak, Thai-Burma border | 28  [17-38] | 21^+5^  [6^+1^-31^+4^] | Fever and headache, joint pain and myalgia | RDT & paired Indirect IFA and /or PCR | Briefly in and detailed | Briefly in and detailed in current manuscript |
| 2014  Sengupta et al | Cohort  N=42 | Vellore, South India | 25  [20-33] | 28  [6-38] | Fever (n=42) | Single *O. tsutsugamushi* IgM ELISA System (Inbios). | Azithro or azithro combination  (n=38) | 1 maternal death; a high rate pregnancy loss 33% (14/42) |

*Abbreviations:Doxy doxycycline; EGA estimated gestational age(weeks); Erythro erythromycin; Genta gentamycin; LBW low birth weight; N.A Not Available; PTB preterm birth*

**REFERENCES**

1. Graves SR, Banks J, Dwyer B, King GK (1992) A case of murine typhus in Queensland. Med J Aust 156: 650-651.

2. Koliou M, Christoforou C, Soteriades ES (2007) Murine typhus in pregnancy: a case report from Cyprus. Scand J Infect Dis 39: 625-628.

3. Gutierrez L, Surani S, Nelson D, El-Milady N (2010) Murine typhus in pregnancy. Am J Med Sci 339: 378-379.

4. Jolley JA, Pelayo R, Hatfield TJ, McNulty J (2010) Murine typhus in a pregnant woman. Obstet Gynecol 116 Suppl 2: 541-543.

5. McGready R, Ashley EA, Wuthiekanun V, Tan SO, Pimanpanarak M, et al. (2010) Arthropod borne disease: the leading cause of fever in pregnancy on the Thai-Burmese border. PLoS Negl Trop Dis 4: e888.

6. Anstey NM, Tissot Dupont H, Hahn CG, Mwaikambo ED, McDonald MI, et al. (1997) Seroepidemiology of Rickettsia typhi, spotted fever group rickettsiae, and Coxiella burnetti infection in pregnant women from urban Tanzania. Am J Trop Med Hyg 57: 187-189.

7. Shirai A, Tanskul PL, Andre RG, Dohany AL, Huxsoll DL (1981) Rickettsia tsutsugamushi strains found in chiggers collected in Thailand. Southeast Asian J Trop Med Public Health 12: 1-6.

8. Suntharasaj T, Janjindamai W, Krisanapan S (1997) Pregnancy with scrub typhus and vertical transmission: a case report. J Obstet Gynaecol Res 23: 75-78.

9. Tsui MS, Fang RC, Su YM, Li YT, Lin HM, et al. (1992) Scrub typhus and pregnancy: a case report and literature review. Zhonghua Yi Xue Za Zhi (Taipei) 49: 61-63.

10. Choi EK, Pai H (1998) Azithromycin therapy for scrub typhus during pregnancy. Clin Infect Dis 27: 1538-1539.

11. Watt G, Kantipong P, Jongsakul K, Watcharapichat P, Phulsuksombati D (1999) Azithromycin activities against Orientia tsutsugamushi strains isolated in cases of scrub typhus in Northern Thailand. Antimicrob Agents Chemother 43: 2817-2818.

12. Mathai E, Rolain JM, Verghese L, Mathai M, Jasper P, et al. (2003) Case reports: scrub typhus during pregnancy in India. Trans R Soc Trop Med Hyg 97: 570-572.

13. Phupong V, Srettakraikul K (2004) Scrub typhus during pregnancy: a case report and review of the literature. Southeast Asian J Trop Med Public Health 35: 358-360.

14. Kim YS, Lee HJ, Chang M, Son SK, Rhee YE, et al. (2006) Scrub typhus during pregnancy and its treatment: a case series and review of the literature. Am J Trop Med Hyg 75: 955-959.

15. Mahajan SK, Rolain JM, Kashyap R, Gupta D, Thakur S, et al. (2009) Scrub typhus complicating pregnancy. J Assoc Physicians India 57: 720-721.

16. Sengupta M, Benjamin S, Prakash JA (2014) Scrub typhus continues to be a threat in pregnancy. Int J Gynaecol Obstet.
